# Estrogen receptors and estetrol-dependent neuroprotective actions: a pilot study

**DOI:** 10.1530/JOE-16-0434

**Published:** 2016-11-17

**Authors:** Ekaterine Tskitishvili, Christel Pequeux, Carine Munaut, Renaud Viellevoye, Michelle Nisolle, Agnes Noël, Jean-Michel Foidart

**Affiliations:** 1 Department of Obstetrics and Gynecology/Department of Clinical SciencesLaboratory of Development Biology and Tumor, GIGA-Cancer, University of Liege, Liege, Belgium; 2 Department of PediatricsNeonatal Intensive Care Unit, University of Liege, CHR de la CITADELLE, Liege, Belgium; 3 Department of Obstetrics and GynecologyUniversity of Liege, CHR de la CITADELLE, Liege, Belgium

**Keywords:** hypoxic–ischemic encephalopathy, estetrol, ERα, ERβ, myelination

## Abstract

Estetrol (E4) has strong antioxidative, neurogenic and angiogenic effects in neural system resulting in the attenuation of neonatal hypoxic–ischemic encephalopathy. We aimed to define the role of estrogen receptors in E4-dependent actions in neuronal cell cultures and prove the promyelinating effect of E4. *In vitro* the antioxidative and cell survival/proliferating effects of E4 on H_2_O_2_-induced oxidative stress in primary hippocampal cell cultures were studied using different combinations of specific inhibitors for ERα (MPP dihydrochloride), ERβ (PHTTP), GPR30 (G15) and palmytoilation (2-BR). LDH activity and cell survival assays were performed. *In vivo* the promyelinating role of different concentrations of E4 (1 mg/kg/day, 5 mg/kg/day, 10 mg/kg/day, 50 mg/kg/day) was investigated using the hypoxic–ischemic brain damage model in the 7-day-old immature rats before/after the induction of hypoxic–ischemic insult. Myelin basic protein (MBP) immunostaining was performed on brain coronal sections. Our results show that LDH activity is significantly upregulated in cell cultures where the E4’s effect was completely blocked by concomitant treatment either with ERα and ERβ inhibitors (MPP and PHTPP, respectively), or ERα and ERβ inhibitors combined with 2-BR. Cell survival is significantly downregulated in cell cultures where the effect of E4 was blocked by ERβ inhibitor (PHTTP) alone. The blockage of GRP30 receptor did affect neither LDH activity nor cell survival. MBP immunostaining is significantly upregulated in E4-pretreated groups at a concentration of 5 mg/kg/day and 50 mg/kg/day E4, whereas the MBP-positive area OD ratio is significantly increased in all the E4-treated groups. E4’s antioxidative actions mostly depend on ERα and ERβ, whereas neurogenesis and possibly promyelinating activities might be realized through ERβ.

## Introduction

Neonatal encephalopathy is mainly triggered by perinatal hypoxic–ischemic brain injury and accompanied by neurodevelopmental deficits such as learning disabilities, mental retardation and hearing and visual impairments. Neonatal hypoxic–ischemic encephalopathy (HIE) remains a serious condition that causes significant mortality and morbidity in near-term and term newborns; also, it does occur in premature infants as well (Volpe 2001). The brain damage due to hypoxia and ischemia results in various lesions in preterm and in term infants with the neuronal/axonal involvement of the cerebral white matter, thalamus, basal ganglia, cerebral cortex, brainstem and cerebellum (Volpe 2001). Two recent clinical trials provided updated information on mortality and neurodevelopmental outcomes in infants with moderate and severe HIE as follows: 23–27% of infant mortality was recorded prior to discharge from the neonatal intensive care unit (NICU) and 37–38% of mortality at follow-up 18–22 months later (Gluckman *et al*. 2005, Shankaran *et al*. 2005). The neurodevelopmental outcome at 18 months included mental and psychomotor development retardation, cerebral palsy (CP), epilepsy, blindness and hearing impairment (Gluckman *et al*. 2005, Shankaran *et al*. 2005). As a recent study suggests, neonatal HIE might start antenatally, implying the importance of different factors (i.e. genetic and/or infectious, and placental factors), but parturition might have importance for the final development of HIE (Martinez-Biarge *et al*. 2013). As a consequence, brain hypoxia and ischemia due to systemic hypoxemia and reduced cerebral blood flow (CBF) are primary reasons leading to perinatal HIE (Grow & Barks 2002, Ferriero 2004). At present, therapeutic hypothermia is considered the best neuroprotective strategy (Gluckman *et al*. 2005, Shankaran *et al*. 2005, Azzopardi *et al*. 2009), but neurodevelopmental deficits persist in 40–50% of patients even after hypothermia (Shankaran *et al*. 2005). So far, no medical treatment provides important neuroprotection against neonatal HIE.

E4 is a natural human fetal estrogen with selective estrogen receptor modulator activity (SERM) (Abot *et al*. 2014). Its synthesis amounts to 1 mg/kg/day at term of pregnancy and results of a cooperativity between fetal adrenals, placenta and fetal liver. It is detected in maternal urine from about 9 weeks of gestation, substantially increasing during pregnancy (Holinka *et al*. 2008). Our recent studies already showed that E4 has very good antioxidant, neuroprotective, neurogenic and angiogenic properties and the combined use of E4 with other steroids do not have any priority over the single use of E4 (Tskitishvili *et al*. 2014, 2016).

Estrogen receptor α (ERα) and β (ERβ) are expressed in the human cortex and hippocampus during neurodevelopment. ERα, detected by 9 weeks of gestation, plausibly has importance for the early neurodevelopment, whereas ERβ might have importance for later processes, such as corticogenesis (Nomura *et al*. 2003). E4 acts as a SERM by activating the nuclear ERα, inhibiting its membrane form and blocking the membrane initiated steroid signaling by estradiol. Depending on the respective role of nuclear and membrane forms of ERα in distinct target organs, E4 may have a synergistic role with E2 (through activation of nuclear ERα) or an anti-estrogenic effect by blocking membrane ERα and its activation by E2. As a consequence, E4 has biological activities distinct from E2, depending on the tissues and cells and the selective binding to the nuclear/membrane form of ERα (Abot *et al*. 2014). In general, palmitoylation regulates 17β-estradiol-induced ERα degradation and transcriptional activity (La Rosa *et al*. 2012) and may explain the ability of ERα to associate to plasma membrane making possible E2-dependent rapid functions (Acconcia *et al*. 2004) and the same might be plausible for E4-dependent rapid functions.

Recent studies have shown that estrogen receptor ERβ expression in oligodendrocytes is required for the attenuation of clinical disease by an ERβ ligand pointing out the role of this specific receptor in myelination (Khalaj *et al*. 2013). It was also demonstrated that GPR30, which is uniquely localized to the endoplasmic reticulum, but not the plasma membrane, may non-genomically signal in response to estrogens by increasing the calcium flux (Revankar *et al*. 2005).

Our aim was to identify those estrogen receptors through which E4 can realize its antioxidative and neuroprotective actions in neuronal cells and define the possible promyelinating effect of E4.

## Materials and methods

### *In vitro* studies

#### Preparation of primary hippocampal neuronal cultures

We prepared primary hippocampal neuronal cultures from newborn (P0) Sprague–Dawley (SD) rat pup brains according to the recently published protocols (Kaech & Banker 2006, Beaudoin *et al*. 2012), which we have used in our previous study (Tskitishvili *et al*. 2014, 2016). Briefly, brains were dissected to separate the hippocampus region. Hippocampi were separated in dissection medium consisting of Hanks balanced salt solution (HBSS) supplemented with sodium pyruvate (100×), glucose and HEPES buffer (10 mM), and trypsinized followed by the addition of DNase and incubation at room temperature for 5 min. Then hippocampi were resuspended in 2.5 mL of plating medium consisting of minimal essential medium (MEM) with Earle’s salts, supplemented with 10% of fetal bovine serum (FBS), glucose, sodium pyruvate, GlutaMax-I-supplement and penicillin/streptomycin (100×). Hippocampi were dissociated, the cell viability was evaluated and the cells were plated on poly-l-lysine-coated 24-well (5 × 10^4^ cells/well) or 96-well (5 × 10^3^ cells/well) culture plates. Cultures were incubated in a humidified 5% CO_2_/95% air atmosphere at 37°C in maintenance medium consisting of neurobasal medium, containing supplement B-27 (50×), GlutaMax-I-supplement and penicillin/streptomycin (100×). Cytosine arabinosidase (Ara-C) was added to the maintenance media 48 h later after plating of the cells during 24 h. Upon changing the culture medium, the cultures were incubated for additional 3–4 days prior to use. All the chemicals and solutions were purchased from Invitrogen and Sigma-Aldrich.

#### Cell culture stimulation with H_2_O_2_, E4 alone or with different combinations of inhibitors

To study the role of different estrogen receptors that mediate the antioxidant effect of E4, specific inhibitors for ER-α, ER-β, GPR30 and palmitoylation (MPP dihydrochloride, PHTTP, G15 and 2-bromohexadecanoic acid (2-BR), respectively) were used as their efficacy was already described in some recent studies (Kajta *et al*. 2013, Rzemieniec *et al*. 2015). According to these studies, inhibitors might give unspecific effects in cell cultures by affecting the LDH release. To avoid any unspecific reaction, effects of different doses of MPP, PHTTP, G15 (from 1 pM to 1 μM of each inhibitor) and 2-BR (1 pM–40 μM) alone or in combination with each other in the presence or absence of either E4 or H_2_O_2_ were tested on LDH release (data not shown). Based on these observations, we have chosen the doses of inhibitors, which did not give unspecific effects and do not affect the LDH release (1 pM of each inhibitor). MPP has no stimulatory activity on ERα or ERβ, and it fully inhibits ERα activity by E2 while having no suppressive activity on ERβ stimulation by E2 (Sun *et al*. 2002). PHTTP has 36-fold selectivity for ERβ, and it is fully effective as an ERβ-antagonist while exhibiting no significant agonist effects on ERα or ERβ. Thus, it is useful in evaluating the biological activity of ERβ (Compton *et al*. 2004). G15 binds to GPR30 with high affinity and acts as an antagonist of estrogen signaling through GPR30 (Dennis *et al*. 2009). 2-BR is a non-selective inhibitor of lipid metabolism and a general inhibitor of protein S-palmitoylation (Davda *et al*. 2013).

MPP dihydrochloride, PHTTP and G15 were purchased from Tocris Bioscience (Bristol, UK), whereas 2-BR – from Sigma-Aldrich. All inhibitors were dissolved in DMSO and further diluted in culture medium at a final concentration of 0.1% DMSO. Primary hippocampal cell cultures prepared from newborn rat pups, at day 7 after plating cells, were treated with 100 µM of H_2_O_2_ for 30 min (Merck KGaA) and then treated with E4 alone or in combination with MPP (1 pM), or PHTTP (1 pM), or G15 (1 pM) and/or 2-BR (1 pM) for 1 h as follows: E4 + 1 pM MPP, E4 + 1 pM PHTTP, E4 + 1 pM MPP + 1 pM PHTTP, E4 + 1 pM 2-BR; E4 + 1 pM 2-BR + 1 pM MPP, E4 + 1 pM2-BR + 1 pMMPP + 1 pMPHTTP, E4 + 1 pM G15. Cell cultures treated only with 100 µM of H_2_O_2_ for 1 h 30 min were used as controls. E4 (Mithra Pharmaceuticals, Liege, Belgium) was used at a concentration of 3.25 mM as one of the successful concentrations used in our previous *in vitro* studies (Tskitishvili *et al*. 2014, 2016). LDH activity was measured in supernatants. The rest of the cell cultures were subjected to the cell viability assay.

#### Evaluation of lactate dehydrogenase (LDH) activity and cell viability

To evaluate the consequence of existence of oxidative stress and the cell viability in primary hippocampal cell cultures stimulated by different concentrations of E4 alone or in combination with different estrogen receptor inhibitors, LDH activity (Abcam) and cell viability assays (Promega Corporation) were performed. All the procedures were performed in accordance to the manufacturer’s protocol as previously described (Tskitishvili *et al*. 2014, 2016). Each condition was repeated 3–6 times.

### *In vivo* studies

We obtained SD pregnant rats from Janvier (Janvier Labs, Le Genest-Saint-Isle, France). After delivery, the newborn pups were reared with their dams at 25°C. All experimental procedures were approved by the University of Liege (Belgium) Ethical Committee. E4 was diluted in saline solution. The vehicle group animals were IP injected a saline solution. Neither injections nor the carotid artery ligation and exposure to hypoxia were performed in sham group.

#### Neuroprotective (pretreatment) effect of E4

To study the neuroprotective effect of E4, 10 newborn rat pups were assigned to each group from postnatal day 4 as followed: sham group, vehicle-treated group, 1 mg/kg/per day, 5 mg/kg/per day, 10 mg/kg/per day and 50 mg/kg/per day E4-treated groups. From postnatal day 4 (P4), pups were injected IP either with vehicle (vehicle group) or with E4 (1 mg/kg/day, 5 mg/kg/day, 10 mg/kg/day or 50 mg/kg/day) in accordance to the group assignment. At postnatal day 7 (P7), a model of hypoxia–ischemia in immature 7-day-old rat pups was used with modifications (Vanucci & Vanucci 2005). Briefly, 30 min after the last injection of either E4 or vehicle, animals were anesthetized with isoflurane (induction 3.0%, maintenance-1.50%), and the left common carotid artery was double-ligated and severed. After the procedure, the pups were returned to their dams and allowed to recover for 1 h. The pups were then placed in the humidified hypoxic *in vivo* cabinet (CoyLab, Grass Lake, MI, USA). Hypoxia was produced by the inhalation of decreased concentrations of oxygen for 20 min from 11% to 8% oxygen balanced by nitrogen, followed by the inhalation of 8% oxygen and 92% nitrogen for 35 min as already described previously (Tskitishvili *et al*. 2014, 2016). All manipulations were performed at 37°C. Rat pups recovered with their dams and reared normally until being killed at P14.

#### Therapeutic effect of E4

To study the therapeutic effect of E4 after hypoxic–ischemic insult, 10 newborn rat pups were assigned to each group at P7 as followed: sham group, vehicle-treated group, 1 mg/kg/per day, 5 mg/kg/per day, 10 mg/kg/per day and 50 mg/kg/per day E4 groups. At P7, a model of hypoxia–ischemia in immature 7-day-old rat pups was used with modifications (Vanucci & Vanucci 2005). Briefly, animals were anesthetized with isoflurane (induction 3.0% maintenance-1.5.0%) and the left common carotid artery was double-ligated and severed in the rat pups of the vehicle and E4 groups. After the procedure, the pups were returned to their dams allowed to recover for 1 h and then placed in the humidified hypoxic *in vivo* cabinet (CoyLab). Hypoxia was produced by the inhalation of decreasing concentrations of oxygen for 20 min from 11% to 8% balanced by nitrogen, followed by inhalation of 8% oxygen and 92% nitrogen for 35 min. All manipulations were performed at 37°C as already described previously (Tskitishvili *et al*. 2014, 2016). Upon retrieval from hypoxia chamber, rat pups were injected IP either by vehicle (vehicle group) or by E4 (1 mg/kg/day, 5 mg/kg/day, 10 mg/kg/day or 50 mg/kg/day) in accordance to the group assignment. Rat pups recovered with their dams until being killed at P14.

#### Measurement of rat pups rectal temperature

To determine the possible effect of E4 treatment on rat pups body and brain temperatures, the measurement of the temperature per rectum was done by using multiple thermometer (BAT-10R) and a specific RET-4 probe (Bio Medical Instruments, Zollnitz, Germany) after hypoxic insult at 0-, 2- and 4-h time points as described previously (Tskitishvili *et al*. 2014, 2016). The variability of the rectal/body temperature was kept at low level by making the temperature measurements in a 25°C room (Feng *et al*. 2005). Rat pups rectal temperature was not significantly different between the study groups (data not shown).

#### Brain and blood samples preparation

The pups were killed at P14. Preparation of the brain and blood samples was performed according to our protocol already used previously (Tskitishvili *et al*. 2014, 2016). Briefly, animals were deeply anesthetized. Blood was withdrawn quickly and the serum samples were stored at −80°C before being used. Transcardial perfusion of animals was performed with 0.9% saline solution followed by the perfusion of the 4% paraformaldehyde in PBS at 4°C. The brains were being quickly removed, weighed and fixed for 24 h, followed by embedment in paraffin.

#### ELISA to detect blood serum S100B and glial fibrillary acidic protein (GFAP) (brain damage markers)

ELISAs for serum S100B (CUSABIO BIOTECH Co., Ltd., Wuhan, China), and GFAP (USCN Life Science Inc., Wuhan, China) were performed according to the manufacturers’ recommendations.

#### MBP staining

The sections were processed for immunohistochemical detection of neuronal cytoskeletal disruption. For antigen retrieval, the sections were heated in 10 mmol/L citrate buffer (pH 6.0) at 100°C for 10 min. Endogenous peroxidase activity was blocked with 3% hydrogen peroxide for 10 min and after a second blocking with 5% normal goat serum, the sections were incubated with MBP 1:1000 (mouse monoclonal antibody; Sigma) overnight at room temperature. After rinsing, biotinylated goat anti-mouse immunoglobulin G (Vector, Burlingame, CA, USA) was added, and antibody detection was performed with the avidin–biotin complex method (Vector), with 3,30-diaminobenzidine (DAB) and the nickel as chromogens. After the reaction with DAB and the nickel, the slides were washed, dehydrated and coverslipped. The area with intact white matter displayed staining with MBP, whereas the damaged area showed a loss of MBP staining. 10 samples from each study group of both study designs were analyzed with the aid of an image scanner (Nanozoomer Virtual Microscopy, Hamamatsu, Tokyo, Japan) and the ImageJ software (NIH) as previously described (Tskitishvili *et al*. 2014, 2016). The optical density (OD) of MBP-positive areas in the ipsilateral and contralateral hemispheres was measured. The ratio of the MBP-positive areas OD was calculated as the MBP-positive area OD of the ipsilateral hemisphere divided by the MBP-positive area of the contralateral hemisphere. The ratio of the MBP-positive area OD in sham-operated animal group was considered by default as 1.0.

#### Correlation studies between the optical density (OD) of MBP-positive area ratio, brain damage markers, glial fibrillary acidic protein (GFAP) and S100B and the brain weight

Previous studies already demonstrated that the reduced brain weight is usually attributed mainly to the loss of white matter (Harper *et al*. 1985, De la Monte 1988), and there is a relationship between expression patterns of myelin protein levels and brain weight, age at death and postmortem interval (Lewoh *et al*. 2005). Recently, a significant correlation was found between the markers for myelination and the brain weight (Lewoh *et al*. 2005). Brain weights of animals from study groups were measured along with the MBP-positive area OD ratio, the blood GFAP and S100B expression, and correlation studies were performed.

### Statistical analysis

The Statview statistics package (Abacus Concepts) was used for statistical analysis. An ANOVA followed by Fisher’s PLSD, Scheffe’s and Bonferroni/Dunn *post hoc* tests and Fisher’s *r* to *z* tests, respectively, were used for statistical comparisons and correlative studies with *P* ≤ 0.05 considered as significant. All values (except for correlations) are expressed as mean ± s.e.m.

## Results

### Effect of E4 treatment with estrogen receptor inhibitors on H_2_O_2_-induced LDH activity in primary hippocampal cell cultures

**Figure 1:**
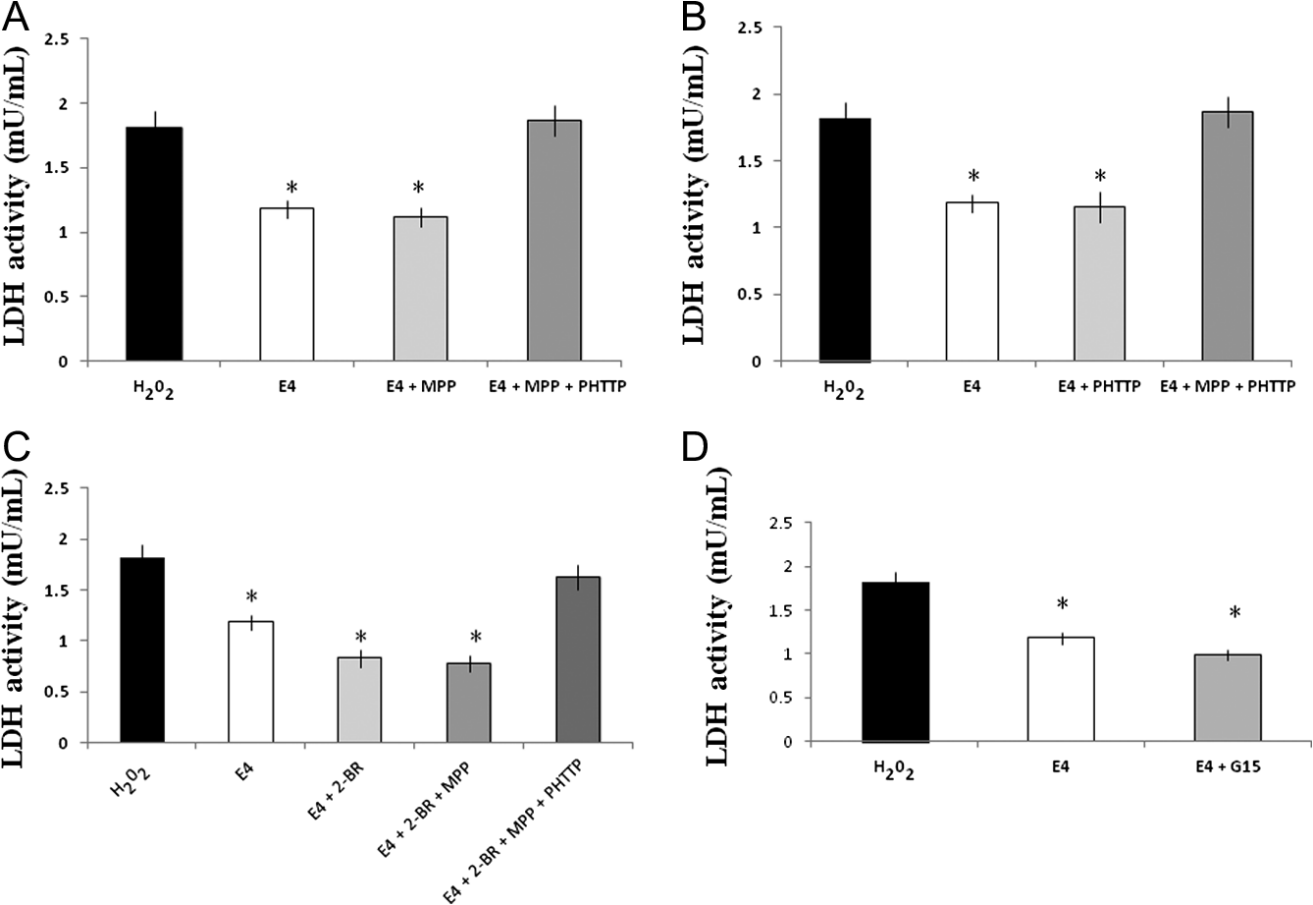
Effect of E4 in combination with different receptor inhibitors on LDH activity in primary hippocampal neuronal cultures subjected to the H_2_O_2_-induced oxidative stress. Primary hippocampal cell cultures were exposed to 3.25 mM E4 alone or in combination with MPP, PHTTP, G15 and/or 2-BR after induction of oxidative stress. (A) LDH activity was significantly decreased by treatment with E4 alone or in combination with ER-α inhibitor MPP compared to the H_2_O_2_-treated cell cultures or cultures combinedly treated by E4 + MPP + PHTTP. Combined use of MPP and PHTTP significantly increased the LDH activity compared to the cells treated by E4 alone or in combination with MPP. (B) LDH activity was significantly decreased by treatment with E4 alone or in combination with ER-β inhibitor PHTTP compared to the H_2_O_2_-treated cell cultures or cultures combinedly treated by E4 + MPP + PHTTP. Combined use of MPP and PHTTP significantly increased the LDH compared to the cell cultures treated by E4 alone or in combination with PHTTP. (C) Inhibition of palmitoylation alone or in combination with MPP significantly downregulated LDH activity compared to the H_2_O_2_-treated cells or to those treated by E4 alone. Combination of E4 with 2-BR, MPP and PHTTP significantly upregulated LDH activity compared to the cell cultures treated by E4 or 2-BR alone or in combination with MPP. (D) Cell cultures treated by E4 alone or in combination with GPR30 inhibitor G15 had significantly lower LDH activity compared to the cultures treated by H_2_O_2_ alone. No significant difference was observed between the cells treated by E4 alone or in combination with G15.

To study the role of estrogen receptors in E4-dependent antioxidative effects in neuronal cells, primary hippocampal cell cultures were exposed to 3.25 mM E4 alone or in combination with different estrogen receptor inhibitors and/or palmitoylation inhibitor after induction of oxidative stress. As shown in Fig. 1A, the LDH activity level was significantly decreased by treatment with E4 alone or in combination with MPP, and only concomitant treatment of cells by E4 along with MPP and PHTTP blocked the antioxidative activity of E4 and significantly increased the LDH activity level compared to the cell cultures treated by E4 alone. Similar pattern of activities was observed when E4 was used in combination with PHTTP alone, and only the combined use of E4 with MPP and PHTTP completely blocked the E4-dependent effect by increasing the LDH activity to the same levels than that in the H_2_O_2_-treated cells (Fig. 1B). As shown in Fig. 1C, inhibition of palmitoylation alone (E4 + 2-BR group) or in combination with MPP significantly decreased LDH activity, suggesting that the combined blockage of ERα and palmitoylation is not sufficient to inhibit the E4-dependent effects. In the presence of 2-BR, the LDH activity was significantly lower than that in H_2_O_2_-treated cell cultures, also in those treated by E4 alone, suggesting that the inhibition of palmitoylation even potentiated the E4-dependent antioxidative effect. Finally, combination of E4 with 2-BR, MPP and PHTTP completely blocked the antioxidative effects of E4 compared to the use of E4 or 2-BR alone or in combination with MPP once again suggesting the role of both receptors, ERα and ERβ (Fig. 1C). Inhibition of GPR30 receptor did not block the E4 actions resulting in significant decrease of LDH activity compared to the cell cultures treated solely by H_2_O_2_ (Fig. 1D).

### Effect of E4 treatment with estrogen receptor inhibitors on H_2_O_2_-induced cell viability in primary hippocampal cell cultures

**Figure 2:**
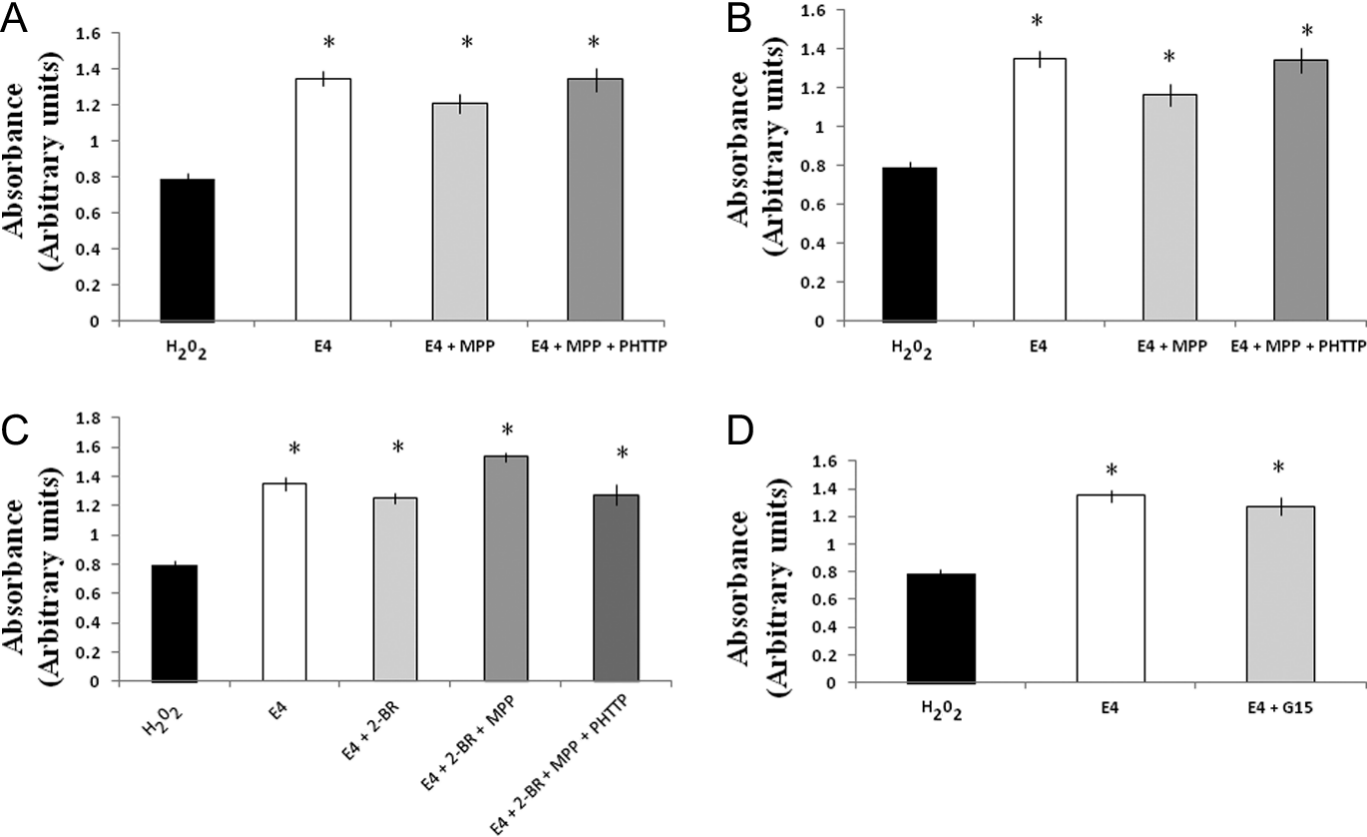
Effect of E4 in combination with different receptor inhibitors on cell survival in primary hippocampal neuronal cultures subjected to the H_2_O_2_-induced oxidative stress. Primary hippocampal cell cultures were exposed to 3.25 mM E4 alone or in combination with MPP, PHTTP, G15 and/or 2-BR after induction of oxidative stress. (A) Cell survival rate was significantly upregulated in cells treated by E4 alone or in combination either with MPP or MPP + PHTTP compared to cells solely treated by H_2_O_2_. (B) Cultures treated either by E4 alone or with PHTTP with/without MPP had significantly upregulated cell survival rate compared to cells treated by H_2_O_2_ alone. Cells combinedly treated by E4 with PHTTP had significantly lower cell survival rate than the cell cultures treated by E4 alone. (C) Cells treated either by E4 alone or in combination with 2-BR, MPP and/or PHTTP had significantly higher cell survival rate compared to the cells solely treated by H_2_O_2_. Treatment of cultures by E4 and 2-BR along with MPP resulted in significant upregulation of cell survival compared to the cultures treated by 2-BR alone or in combination with MPP and PHTTP. No significant difference was observed between the cells treated by E4 alone or those treated by different combinations of E4, 2-BR, MPP and/or PHTTP. (D) Treatment of cell cultures by E4 alone or in combination with G15 significantly upregulated the cell survival rate compared to cell cultures treated by H_2_O_2_. No significant difference was observed between cells treated by E4 alone or in combination with G15.

To study the role of estrogen receptors in E4-dependent cell survival/proliferation effects in neuronal cells, primary hippocampal cell cultures were exposed to 3.25 mM E4 alone or in combination with different estrogen receptor-specific inhibitors and/or palmitoylation inhibitor after induction of oxidative stress. As shown in Fig. 2A, cell survival rate was significantly higher in cells treated by E4 alone or in combination either with MPP alone or when associated with PHTTP suggesting that neither inhibition of ERα alone nor inhibition of both receptors (ERα and ERβ) blocked the E4-dependent cell survival activity. Inhibition of ERβ alone significantly downregulated the cell survival rate compared to cells treated by E4 alone or in combination with MPP and PHTTP (Fig. 2B). Figure 2C demonstrates that all cells treated either by E4 alone or in combination with different combinations of 2-BR, MPP and PHTTP had significantly higher cell survival rate compared to the cells treated by H_2_O_2_ alone, and inhibition of palmitoylation along with inhibition of ERα activity resulted in a significantly higher cell survival compared to the cultures treated by 2-BR alone or in combination with MPP and PHTTP suggesting that ERα (probably membrane form of the receptor) does not affect the E4-dependent cell survival/proliferation actions (Fig. 2C). Combination of E4 with G15 significantly upregulates the cell survival rate along with cell cultures treated by E4 alone compared to the cells treated by H_2_O_2_ alone (Fig. 2D).

### Myelin basic protein staining

**Figure 3:**
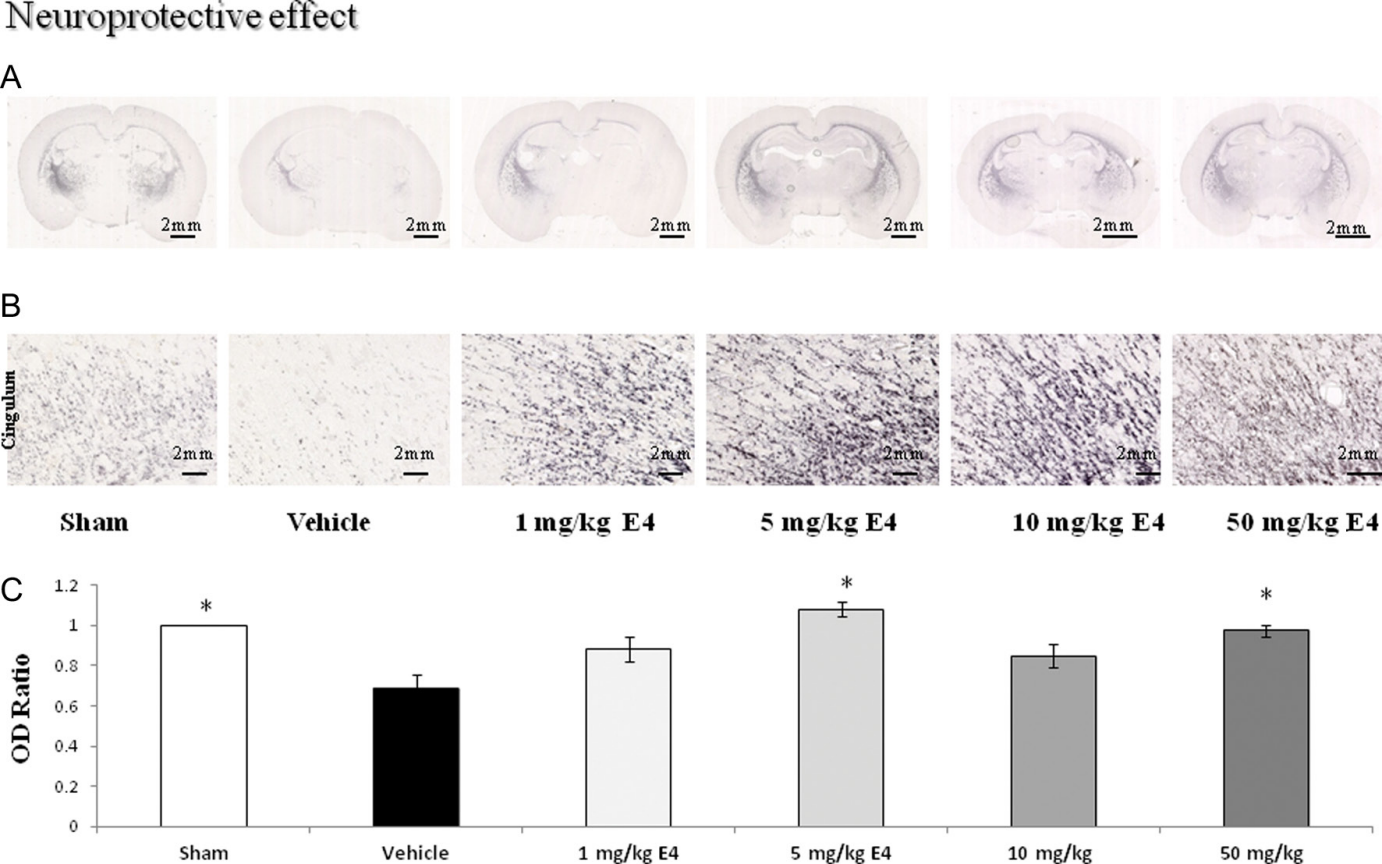
Myelin basic protein (MBP) staining of brain coronal sections in rat pups pretreated with estetrol. (A) MBP staining of brain coronal sections (scale bar: 2 mm) is shown. (B) MBP staining of cingulum of the left hemisphere is shown (scale bar: 2 mm). (C) The ratio of the MBP-positive areas OD ratio was calculated as the MBP-positive area OD of the ipsilateral hemisphere divided by the MBP-positive area OD of the contralateral hemisphere. 10 samples from each study group were analyzed. The ratio of the MBP-positive area OD in the Sham group was considered by default as 1.0. The MBP-positive area OD ratio was significantly higher in sham-operated animals and the 5 mg/kg/day and 50 mg/kg/day E4-pretreated groups compared to the vehicle group. All measurements are expressed as mean ± s.e.m. *P < 0.05.

**Figure 4:**
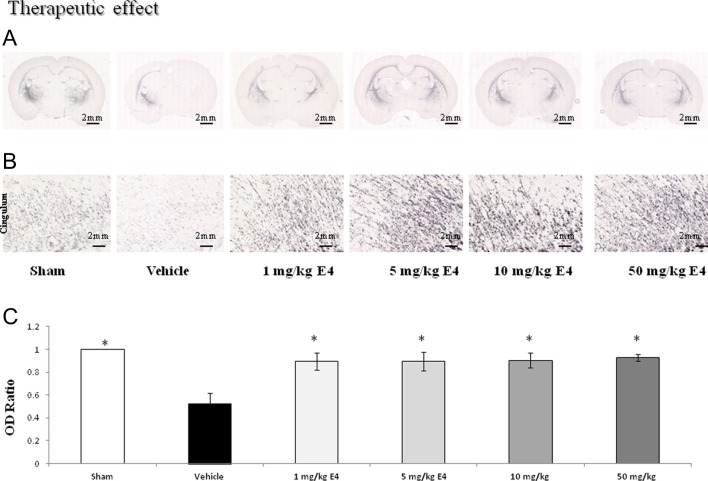
Myelin basic protein (MBP) staining of brain coronal sections in rat pups treated with estetrol. (A) MBP staining of brain coronal sections (scale bar: 2 mm) is shown. (B) MBP staining of cingulum of the left hemisphere is shown (scale bar: 2 mm). (C) The ratio of the MBP-positive areas OD ratio was calculated as the MBP-positive area OD of the ipsilateral hemisphere divided by the MBP-positive area OD of the contralateral hemisphere. 10 samples from each study group were analyzed. The ratio of the MBP-positive area OD in the sham group was considered by default as 1.0. The MBP-positive area OD ratio was significantly higher in sham-operated animals and the 1 mg/kg/day, 5 mg/kg/day, 10 mg/kg/day and 50 mg/kg/day E4-treated groups compared to the vehicle group. All measurements are expressed as mean ± s.e.m. *P < 0.05.

Loss of MBP staining due to hypoxic–ischemic insult was used as a marker of white matter damage. In both study designs, in the vehicle groups, there was a loss of MBP staining in the left hemisphere (Figs 3A and 4A), in the subcortical region and the cingulum as one of the main white matter region of the brain (Figs 3B and 4B). In neuroprotective model, the ratio of MBP-positive area OD was significantly higher in the sham-operated, and in the 5 mg/kg/day and 50 mg/kg/day E4-pretreated groups than that in the vehicle-treated group (Fig. 3C), whereas all the E4-exposed groups in the therapeutic model had significantly higher MBP-positive area OD ratio along with sham group compared to the vehicle group (Fig. 4C).

### Correlation studies between the brain weight, blood GFAP and S100B expression and MBP-positive area OD ratio

To study the significance of the white matter damage marker MBP, and the brain damage markers GFAP and S100B in connection with the brain weight, correlation studies were performed. In most groups from neuroprotective and therapeutic models, no correlation was found between the MBP-positive area OD ratio, GFAP, S100B and the brain weights (data not shown). However, in neuroprotective model, significant positive correlation was observed between the MBP-positive area OD ratio and the brain weights (*r* = 0.707, *P* = 0.0198) in the vehicle group.

## Discussion

In this study, we attempted to identify ER receptors responsible for E4-mediated neuroprotection *in vitro* and evaluate the pro-myelinating efficacy of E4 *in vivo*.

The expression of ERα and ERβ displays different spatial–temporal patterns during human cortical and hippocampal development and suggest that both ERs may play distinct roles in several processes related to prenatal brain development (Gonzalez *et al*. 2007). Knowledge of the region-specific expression of each ER subtype is critical to better understand the actions of estrogens on human brain (Gonzalez *et al*. 2007). The cortical plate, which develops into the future six-layered neocortex, appears first at 8–9 gestational weeks and increases in thickness until neurogenesis is completed after midgestation; however, myelination starts before birth and is not completed in some regions of the brain before adulthood (Feng *et al*. 2005). Highly divergent and sometimes opposing functions for the two receptors have been reported in studies of ERα-knockout and ERβ-knockout mice (Hewitt & Korach 2003). In addition to their effects on gene expression (their genomic effects), these ERs are also associated with rapid cellular signaling (non-genomic effects) that are thought to be mediated primarily by membrane-associated forms of these receptors (Hammes & Levin 2007).

In oligodendrocytes (OLGs) and neuronal cells, ERs are expressed in a different way (Zhang *et al*. 2004). According to recent studies, ERα and ERβ mRNA was detected in OLGs: *in vitro* ERβ is localized in cytoplasm of OLGs, whereas ERα is detected in the nuclei of OLGs. *In vivo* ERβ is detected in cytoplasm and myelin of OLGs, and it is associated with the outer and inner layers of the myelin sheath, whereas in neurons mainly with cytoplasm, and ERα is detected in the nuclei of neurons (Zhang *et al*. 2004). Some recent studies show that the myelin sheath contains an array of proteins and lipids including G proteins (Larocca *et al*. 1991, Dyer 2002, Razandi *et al*. 2003). Classical steroid receptors, localized in the cytosol and/or nucleus, traditionally mediate their primary effects at the genomic level. In recent years, a large number of reports have described membrane-associated estrogen receptors, either similar to or distinct from the classical nuclear estrogen receptors (Toran-Allerand *et al*. 2002, Razandi *et al*. 2003, Acconcia *et al*. 2004). These receptors have been postulated to mediate aspects of cellular estrogen function, including traditional genomic (transcriptional) signaling as well as non-genomic (rapid) signaling (Evans & Muldoon 1991, Govind & Thampan 2003). These non-genomic signaling events include pathways that are traditionally thought of as arising from transmembrane growth factor receptors and G-protein-coupled receptors, whereas some reports described estrogen-binding sites on intracellular membranes (Wang *et al*. 2002, La Rosa *et al*. 2012), other reports suggest that palmitoylation or phosphorylation (La Rosa *et al*. 2012) may target classical ERs to the cytoplasmic side of the plasma membrane. In general, palmitoylation is necessary for ERα transcriptional activity and inhibition of ERα palmitoylation constitutively addresses ERα to the nuclear matrix resulting in the basal degradation of the neo-synthesized ERα (Suzuki *et al*. 2007).

Different studies enlighten the role of estrogen receptors in the central neuronal system (CNS): both estrogen receptors ERα and ERβ play pivotal functional roles, insofar as knocking out either of these receptors blocks the ability of estradiol to increase neurogenesis (Dubal *et al*. 2001, Suzuki *et al*. 2007). ERα mediates protection of the brain and carries the far-reaching implications for the selective targeting of ERs in the treatment and prevention of neural dysfunction associated with normal aging or brain injury (Dubal *et al*. 2001). Our results demonstrate for the first time that only concomitant inhibition of ERα and ERβ receptor activities is necessary to diminish the E4’s antioxidative effects and promote further upregulation of LDH activity suggesting that E4 might be an equally important ligand for both estrogen receptors. One limitation of our study might be connected to failure to prove whether depalmitoylation is connected somehow to the membrane receptor ERα.

Recent studies proved that the neuroprotective actions of estrogens also depend on their strong antioxidant specifications and positively correlate with the number of the phenolic moiety in their structure; existence of the free phenolic OH group is important for protection against oxidative stress (Prokai *et al*. 2006). The highest number of the free phenolic OH groups among estrogens is in E4, suggesting the strong antioxidant effects of this compound. Much research has been done to study the mitochondria as a primary target for estrogen-mediated pathways (Nilsen & Brinton 2004, Nilsen *et al*. 2006, 2007, Brinton 2008, Irwin *et al*. 2008). Our study determines clearly that antioxidative activity of E4 depends on ER activity and is not limited to its ‘dismissal’ capacity to inactivate free oxygen radicals through the OH groups.

As it was defined, the potential role of ERβ expression in cells of oligodendrocyte (OL) lineage in ERβ ligand-mediated neuroprotection is important, and it results in the upregulation of myelination (Khalaj *et al*. 2013). Moreover, neuroprotection might be mediated through ERα in astrocytes exclusively (Spence *et al*. 2011). As we observed, in primary hippocampal cell cultures, the cell survival rate was significantly downregulated only when the ERβ receptor was completely blocked, suggesting the role of ERβ in neurogenesis. We can speculate that promyelinating effects of E4 are also realized through ERβ. We did not observe any significant effect of palmitoylation inhibition on cell survival/proliferation.

The rat begins to form myelin at about 10–12 days postnatally. At 15 days of age, about 4 mg of myelin can be isolated from 1 brain (Siegel *et al*. 1999). This amount increases 6-fold during the next 15 days; and at 6 months of age, 60 mg of myelin can be isolated from 1 brain. This represents an increase of about 1500% over 15-day-old animals. During the same 5.5-month period, the brain weight increases by 50–60% in long-term brain development (Siegel *et al*. 1999). Thus, myelination and the brain weight might be correlated. According to our studies, pretreatment of rat pups by 5 mg/kg/day and 50 mg/kg/day E4 before hypoxic–ischemic injury, and treatment by all E4 doses after HI brain injury significantly upregulates myelination. Correlation studies detected significant positive correlation between the MBP-positive area OD ratio and the brain weights exclusively in the vehicles from the neuroprotective model, whereas in E4-pretreated or -treated groups, this correlation could no longer be observed suggesting that the promyelinating effect of E4 is not the only factor that affects the brain weight in the model of E4-mediated neuroprotection.

In conclusion, E4 is an estrogen with SERM properties. Further studies are necessary to uncover the role of this compound during antenatal neurodevelopment and in attenuation of neonatal hypoxic–ischemic brain damage.

## Declaration of interest

Prof J M Foidart is extraordinary professor at the University of Liege and Senior Scientific officer of Mithra Pharmaceuticals Inc (Belgium).

## Funding

This research was supported by grants from the Fonds de la Recherche Scientifique – FNRS (F.R.S. – FNRS, Belgium): FRSM 3.4557.12, FRSM 3.4526.12, FRSM 3.4567.11, PDR T.0091.14, the Fonds spéciaux de la Recherche (University of Liege, Belgium): FSRC-12/64, FSRC-12/92, FSRC-14/89, FSRC-14/65, FSRC-14/109, FSRC-14/62, the Fonds Léon Fredericq (University of Liege, Belgium), the Direction Générale Opérationnelle de l’Economie, de l’Emploi et de la Recherche from the Service Public de Wallonie (SPW, Belgium): WB Health project No. 1318051, WB Health project No. 1318039, WB Health project No. 1318030, WB Health project No. 1318071,WB Health project No. 1318023, the Interuniversity Attraction Poles Programme—Belgian Science Policy (Brussels, Belgium): IAP Phase VII – P7/03, and the Actions de Recherche Concertées (University of Liege, Belgium): A.R.C. 11/16-02.
